# L-shape triple defects in a phononic crystal for broadband piezoelectric energy harvesting

**DOI:** 10.1186/s40580-022-00321-x

**Published:** 2022-06-15

**Authors:** Soo-Ho Jo, Heonjun Yoon, Yong Chang Shin, Wonjae Choi, Byeng D. Youn, Miso Kim

**Affiliations:** 1 Department of Mechanical Engineering, Seoul National University, Seoul, 08826 Republic of Korea; 2 Institute of Advanced Machines and Design, Seoul National University, Seoul, 08826 Republic of Korea; 3 School of Mechanical Engineering, Soongsil University, Seoul, 06978 Republic of Korea; 4 Intelligent Wave Engineering Team, Korea Research Institute of Standards and Science, Daejeon, 34113 Republic of Korea; 5 OnePredict Inc, Seoul, 06160 Republic of Korea; 6 School of Advanced Materials Science & Engineering, Sungkyunkwan University, Suwon, 16419 Republic of Korea

**Keywords:** Phononic crystal, Band gap, Multiple defects, Defect band, Energy localization, Piezoelectric energy harvesting, Broadband

## Abstract

This study proposes a phononic crystal (PnC) with triple defects in an L-shape arrangement for broadband piezoelectric energy harvesting (PEH). The incorporation of defects in PnCs has attracted significant attention in PEH fields owing to properties such as energy localization and amplification near the defect. Several studies have been conducted to enhance output electric power of PnC-based PEH systems with single defects. However, it is susceptible to the limitations of narrow bandwidth. Recently, double-defect-incorporated systems have been proposed to widen the PEH bandwidth via defect-band splitting. Nevertheless, the PEH performance rapidly decreases in the frequency range between the split defect bands. The limitations of single- and double-defect-incorporated systems can be resolved by the incorporation of the proposed design concept, called the L-shape triple defects in a PnC. The isolated single defect at the top vertex of the letter ‘L’ compensates for the limitations of double-defect-incorporated systems, whereas the double defects at the bottom vertices compensate for the limitations of the single-defect-incorporated systems. Hence, the proposed design can effectively confine and harvest elastic-wave energy over broadband frequencies while enhancing the application of single and double defects. The effectiveness of the proposed design concept is numerically validated using the finite element method. In the case of a circular hole-type PnC, it is verified that the PnC with L-shape triple defects broadens the bandwidth, and improves the output voltage and electric power compared with those of single- and double-defect-incorporated systems. This study expands the design space of defect-incorporated PnCs and might shed light on other engineering applications of the frequency detector and elastic wave power transfer.

## Introduction

Integration of the exceptional wave-manipulating properties of phononic crystals (PnCs) with piezoelectric energy harvesting (PEH) systems has led to the enhancement of output electric power through the amplification of the input elastic-wave energy provided to piezoelectric devices [1–4]. The promising PEH technology converts ambient mechanical energy into usable electric energy owing to the direct piezoelectric effect [5–8]. Although conventional PEH has attracted considerable attention owing to its applications in Internet-of-Things [9, 10], its output electric power degrades if the transferred elastic-wave energy is insufficient. Several methods have been proposed to focus the elastic-wave energy on piezoelectric devices via the unusual nature of PnCs.

A PnC [11–14] is an advanced multi-functional structure assembled through the periodic repetition of unit cells that can ensure elastic-wave control in an extraordinary way owing to properties such as gradient-index-based focusing [15], topologically protected guides [16], and negative refraction [17]. In particular, several pioneering researchers have conducted studies to determine the band gap and frequency range that can suppress the elastic-wave-energy transport through the PnCs [18, 19]. Repeated destructive interferences provoked by the periodicity of the unit cells lead to band-gap formation in dispersion relations (i.e., frequency versus wavenumber) [20, 21]. The incorporation of a disordering structure (called a defect) in a PnC through the partial disruption of periodicity can generate characteristics within the band gap, namely, the appearance of flat defect bands [22]. Each defect band exhibits a different energy-localized displacement field of the PnC (called a defect-mode shape) at each defect-band frequency [23, 24], and this property ensures high amplification of the elastic-wave energy within the defect [25–27]. Hence, piezoelectric devices with defects can generate significantly improved electric power.

Several studies have probed related subjects of advanced PEH strategies that use a PnC with a single defect. Jo et al. [28, 29] proposed analytical models that elucidated the piezoelectric-effect-induced defect-band shift. Jo and Youn [30] proposed an explicit solution for a one-dimensional PnC design based on these models that maximized the output voltage at the target frequency. Lv et al. [31] and Park et al. [32] conducted experiments to enhance the output electric power of one- and two-dimensional PnCs. Jo et al. [33] numerically explored the effects of the supercell size and defect location on the PEH performance through a parametric study. Lee et al. [34] experimentally investigated the change in the PEH performance with different geometric dimensions and materials of the piezoelectric device. Lv et al. [35] improved the PEH performance in a low-frequency range by using high contrast characteristics between the inclusion (lead) and matrix (rubber) in the unit-cell design. Carrara et al. [36] reported severe reduction in the output electric power due to voltage cancellation for a particular defect-mode shape. Geng et al. [37, 38] presented the dependence of output performance of PEH on the external temperature.

**Fig. 1:**
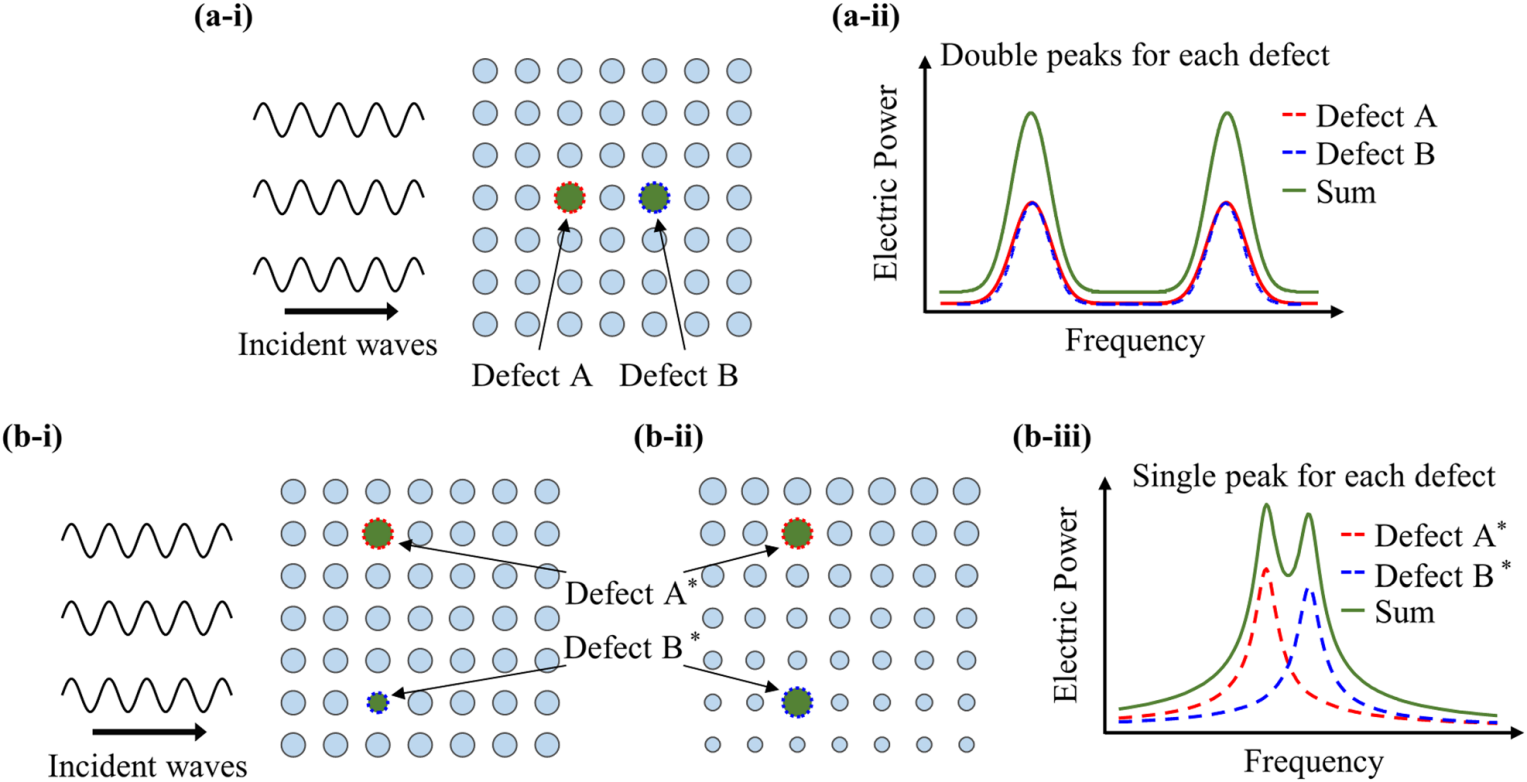
Design concepts of a double-defect-introduced PnC and corresponding output electric power: **a**-**i** a uniform PnC with coupled double defects introduced parallel to incident elastic waves, **a**-**ii** broadband PEH by using defect-band splitting, **b**-**i** a uniform PnC with differently configured & decoupled double defects, **b**-**ii** a graded PnC with identically configured & decoupled double defects, **b**-**iii** broadband PEH by closely placing the defect band of each isolated defect

However, previous studies conducted on piezoelectric devices with single defects have demonstrated intrinsic limitations such as narrow bandwidth of the output electric power due to the utilization of only one defect band. Ma et al. [39] presented the possibility of broadband PEH to address this issue by closely positioning several defect bands of the single defect to which multiple piezoelectric devices were attached. Recently, Jo et al. [40] pushed the boundaries of the PnC design space into double defects. When the double defects are arranged along the direction ‘parallel’ to that of the incident plane waves, the coupling characteristics between the double defects split each defect band obtained from the single defect into two bands as shown in Fig. 1a-i. Figure 1a-ii shows that the PEH performance can be amplified at two peak frequencies with in- and out-of-phase energy-localized behaviors. Here, the in- and out-of-phase behaviors refer to the synchronous motions of the double defects that the amplified displacement fields of each defect are polarized in the same and opposite directions. In this context, the feasibility of broadband PEH via double defect modes has been confirmed. Similarly, Geng et al. [41] investigated the variation in the output electric power of a double-defect-incorporated PnC with external temperature. Conversely, the defects are at a sufficient distance from each other when the double defects are arranged along the direction ‘perpendicular’ to the incident plane waves in a uniform PnC with differently configured and decoupled double defects, (Fig. 1b-i) or in a graded PnC with identically configured and decoupled double defects (Fig. 1b-ii). The weakened interaction between the double defects makes them appear independent. One peak frequency corresponding to each defect is independently manipulated at this point by varying the geometric dimensions of piezoelectric devices attached to each defect [42] or unit cells surrounding each defect [43]. Therefore, closely placing the defect band of each isolated defect successfully doubles the bandwidth of a PEH system as shown in Fig. 1b-iii.

In this study, a design concept, called *a PnC with L-shape triple defects*, is proposed to widen the available bandwidth of the output electric power by combining the ‘parallel’ case with the coupled double defects and the ‘perpendicular’ case with the decoupled double defects. In the case of perpendicular-double-defect, it has been demonstrated that the decoupling nature between the double defects widens the bandwidth by conceptually superimposing two single-defect-incorporated PnCs into one system. Therefore, it is hypothesized that these decoupled properties can amalgamate the parallel-double-defect and perpendicular-double-defect cases for broadband energy localization and harvesting. Such an attempt has not been reported in the literature.

The remaining paper is divided into three sections. The design methodology of the proposed PnC is explained in Sect. 2. Section 3 delineates the results and discussion of the numerical validation studies. Finally, a summary of the study is provided in Sect. 4.

## Proposed phononic crystals with L-shape triple defects

**Fig. 2:**
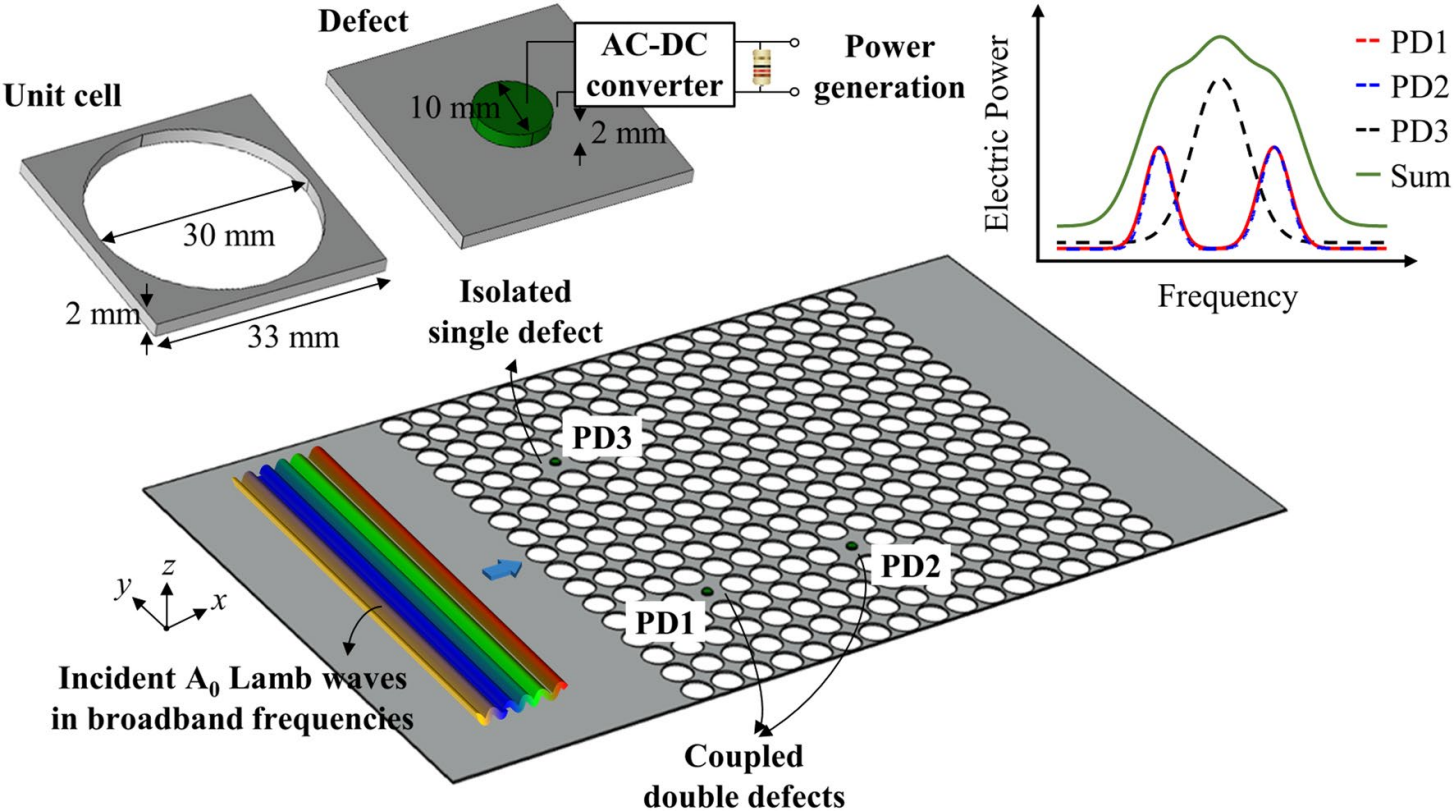
A schematic illustration of the proposed PnC design with L-shape triple defects (PD1, PD2, and PD3) for broadband energy localization and harvesting, relative to right-going incident plane A_0_ Lamb waves

Figure 2 represents a schematic illustration of the proposed PnC with L-shape triple defects. The PnC is incorporated on a thin plate and it consists of a 17 × 17 array of square-lattice-type unit cells. The *x*- and *y*-axes indicate the direction parallel and perpendicular to the incident plane A_0_ Lamb waves normally entering the PnC structure, and the *z*-axis represents the out-of-plane direction. The unit cell refers to a square lattice of aluminum with a circular hole perforated therein. The lattice constant and thickness are 33 and 2 mm. The diameter of the circular hole is 30 mm. The geometric design and material of the unit cell, supercell configuration, and piezoelectric disc are adopted from the previous study in Ref. [40] to compare the proposed and existing double-defect-incorporated PnC systems. It has been stated in Ref. [40] that an arrangement of 17 × 17 periodic unit cells is sufficient to ensure high accuracy of defect-band calculation [44] and exhibit asymptotically converged PEH performance [33]. A defect refers to a raw square lattice of aluminum in the absence of a hole. The defect positioned in the lower-left corner is denoted as piezoelectric defect 1 (PD1) and each defect along the counterclockwise direction is marked as piezoelectric defects 2 (PD2) and 3 (PD3) as shown in Fig. 2. The ordered pair (*m*, *n*) in the top view of the PnC denotes the location of the lattice deployed in the *m*-th column along the *x*-axis and the *n*-th row in the *y*-axis considering the unit cell that is in the bottom left. According to this notation, PD1, PD2, and PD3 are deployed in (4, 5), (9, 5), and (4, 13) respectively. Note that the defect bands depend on the geometric dimensions of the unit cell. Two reasons why the presented values are used as follows. First, the lowest frequency to excite A_0_ Lamb waves in experiments is known to be near 50 kHz [32, 34]. Second, a unit-cell design is preferred in which the target defect bands are distant from the remaining defect bands to clarify the PEH performance.

A disc-type piezoelectric device of PZT-4D (lead zirconate titanate, Pb(Zr_x_Ti_1−x_)O_3_) is attached to the upper surface of the center of the defect for power generation. This composite structure is called a piezoelectric defect. The diameter and height of the piezoelectric disc are 10 and 2 mm. Negligibly thin electrodes layered on the top and bottom sides of the piezoelectric disc are connected to electric circuits composed of an alternating-current-to-direct-current (AC-DC) converter and electrical resistance. Perfect adhesion at all interfaces is assumed to avoid undesirable delamination effects. Table 1 lists the material properties of aluminum and PZT-4D. These values are obtained from a built-in datasheet in Comsol Multiphysics, which is a widely used finite-element-method software [45–47].

**Table 1:** Material properties of the aluminum and PZT-4D

Description	Value
Aluminum	
Density	2700 kg/m^3^
Young’s modulus	70 GPa
Poisson’s ratio	0.33
PZT-4D	
Density	7600 kg/m^3^
Elastic constant at an electric field	
*c*_11_	135 GPa
*c*_12_	98.5 GPa
*c*_13_	93.1 GPa
*c*_33_	128 GPa
*c*_44_	238 GPa
*c*_66_	277 GPa
Piezoelectric constant	
*d*_31_	− 4.73 C/m^2^
*d*_33_	15.3 C/m^2^
*d*_15_	13.1 C/m^2^
Relative dielectric permittivity at constant stress	
*ε*_11_/*ε*_0_	797
*ε*_33_/*ε*_0_	763

Three notable design methodologies are proposed. First, PD1 and PD2 are located on the same row along the *y*-axis. The previous studies conducted on sonic [48] and magnonic [49] crystals included two cases where double defects were arranged in oblique and axial directions. The studies demonstrated that the defect-band splitting in the oblique case was insignificant. This was because the coupling between the obliquely arranged double defects was significantly weaker than that between the double defects parallel to the incident waves. Since the aim of this study is to constructively utilize the defect-band-splitting phenomenon to widen the PEH bandwidth, it is desirable to place PD1 and PD2 in the same row. Second, PD1 and PD3 are located on the same column along the *x*-axis. Since it is assumed that plane waves are normally incident on the PnC, the transferred wave energy do not change along the *y*-axis. According to the previous study [33] which included the effects of *x*-directional defect-location on PEH performance, output electric power varied with the defect location. Therefore, it exhibited the presence of power-optimal position. Hence, it is desirable to determine the power-optimal *x*-axial location where the maximum output electric power can be obtained in the case of a single defect through a parametric study and place PD1 and PD3 in the corresponding *x*-axial layer. According to the preliminary study wherein the unit cell was used, the power-optimal defect location was at the 4th column. Finally, PD2 is positioned close to PD1, while PD3 is placed distant from the PD1 to ensure that a set of PD1 and PD2 becomes coupled, but a set of PD1 and PD3 experiences decoupling. Considering the inter-distance effects of double defects on energy-localization performance in Ref. [50], an increase in the inter-distance between the double defects degraded the coupling between them and reduced the splitting degree of the defect bands. The double defects became decoupled after a particular inter-distance. Therefore, it is crucial to determine the *x*-axial location of PD2 and the *y*-axial location of PD3 relative to PD1 through the parametric study that deals with inter-distance effects of double defects. According to the preliminary study wherein the unit cell was used, it was observed that placing five unit cells between the double defects sufficiently exhibited the decoupling phenomenon. Therefore, four unit cells are present between PD1 and PD2 and seven unit cells are present between PD1 and PD3 in this setting. Recall that a disc-type piezoelectric device is integrated into the proposed PnC and it is attached at the center of respective defects, PD1, PD2, and PD3.

## Result and discussion

### Defect bands and corresponding defect mode shapes

The defect-band results are presented in the band-structure analysis under short-circuit (i.e. the exact zero electrical resistance). Short-circuit means that only mechanical coupling is present between the defect and piezoelectric disc. As a comparison group, the results of the PnC with the single defect located at (4, 9), and the PnC with the coupled double defects located at (4, 9) and (9, 9) are examined. The defect-band results in the single-defect case are the same regardless of the location of the unit cell for the defect imposition. Additionally, the defect-band results are independent of the locations of double defects once the relative position between the double defects is retained.

**Fig. 3:**
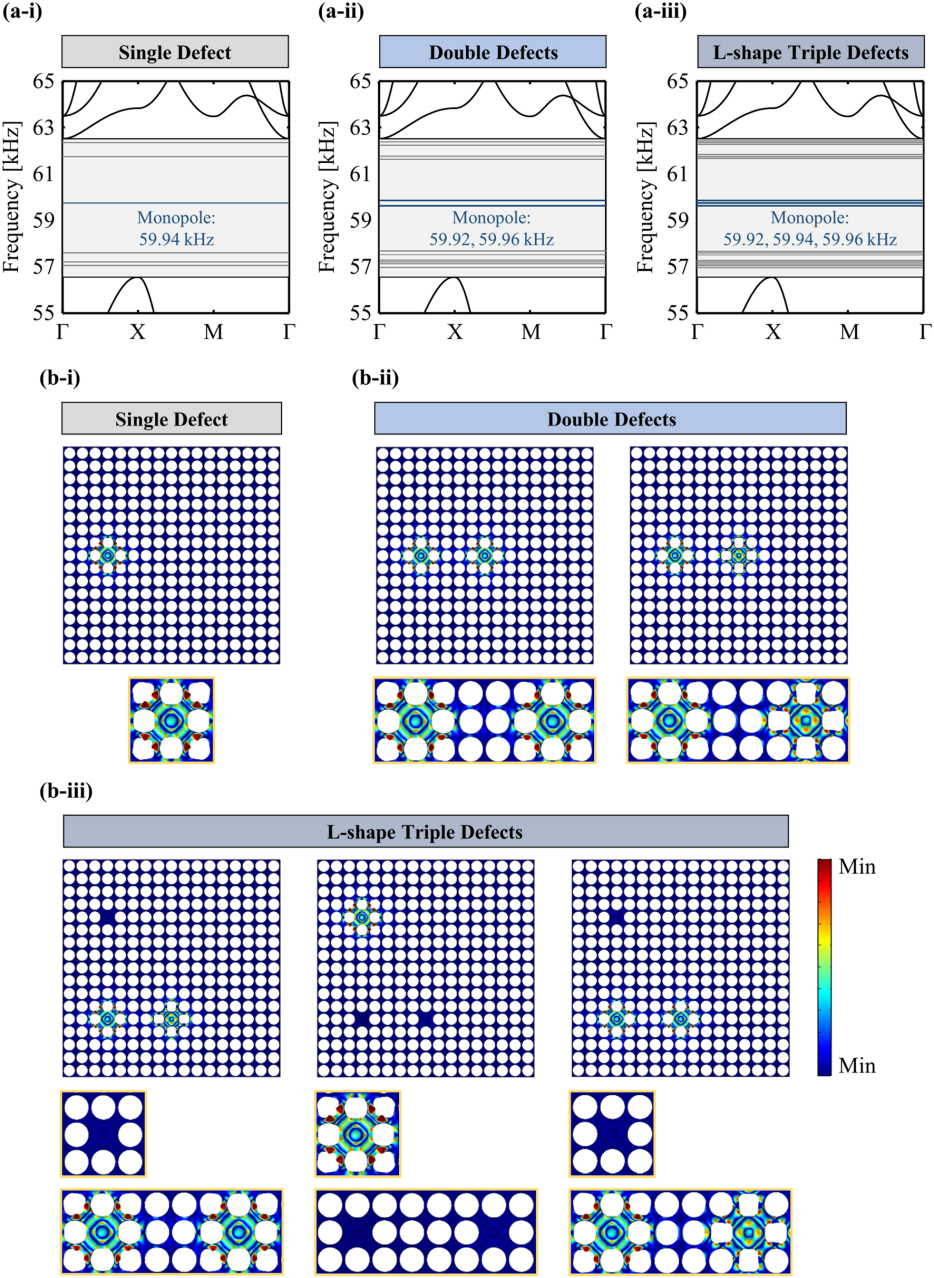
Defect-band results under short-circuit in the band-structure analysis: defect-band formation in the cases of **a**-**i** single defect, **a**-**ii** double defects, and **a**-**iii** the proposed L-shape triple defects, and the monopole-like defect-mode shapes of **b**-**i** single defect, **b**-**ii** double defects, and **b**-**iii** the proposed L-shape triple defects

Figure 3a-i to a-iii show the results of the single defect, double defects, and L-shape triple defects. The band gap (colored in a light gray box) is in the range of 56.55–62.51 kHz in all the cases. Six defect bands can be observed in Fig. 3a-i with defect-band frequencies of 57.04, 57.20, 57.55, 59.94, 61.62, and 62.39 kHz. It can be observed in Fig. 3a-ii that each defect band in the single-defect is separated into two and the corresponding defect-band frequencies are 56.96, 57.11, 57.14, 57.26, 57.53, 57.57, 59.92, 59.96, 61.60, 61.64, 62.38, and 62.40 kHz. In the case of the proposed PnC (Fig. 3a-iii), 18 defect bands are observed and the corresponding frequencies are 56.96, 57.04, 57.11, 57.14, 57.20, 57.26, 57.53, 57.55, 57.57, 59.92, 59.94, 59.96, 61.60, 61.62, 61.64, 62.38, 62.39, and 62.40 kHz. It can be observed that the union of the defect-band frequencies in the single- and double-defect cases is consistent with those in the proposed PnC design case. These results imply the following three conclusions. First, L-triple PD3 is effectively isolated from the remaining L-triple PD1 and L-triple PD2. Therefore, it behaves as a single-defect-incorporated PEH system. Second, L-triple PD1 and L-triple P2 behave as a double-defect-incorporated PEH system. Third, the decoupling nature enhances the spatial superposition of two different systems in the proposed design. Note that the literature review presents that elastic waves over frequencies from tens to hundreds of kHz propagate in transformers [51] and rotating machinery [52]. Therefore, these defect-band frequencies lie in a reasonable range.

Different energy-localized behaviors can be observed in each defect band provided in Fig. 3a-i, and it is required to select one target defect-mode shape. Previous studies conducted on defect-mode-enabled PEH enhancement have demonstrated that the monopole-like defect-mode shape was the most suitable for preventing nodal-line-induced voltage cancellation [36, 53]. Jo et al. [33] revealed that the guideline for the selection was to determine the defect-mode shape that exhibited the maximum increase in the frequency due to the strongest piezoelectric coupling when the electric boundary condition was changed from short-circuit to open-circuit. Open-circuit (i.e., a very large electrical resistance) means that the piezoelectric disc can generate the maximum output voltage due to piezoelectric coupling. In this setting, the defect band of 59.94 kHz in the single-defect case, and the defect bands of 59.92 and 59.96 kHz in the double-defect case are selected. The corresponding defect-mode shapes are shown in Fig. 3b-i and b-ii. The remaining defect-mode shapes are mentioned in Ref. [40]. Figure 3b-iii depicts the defect-mode shapes of the proposed PnC design at defect-band frequencies of 59.92, 59.94, and 59.96 kHz. Only L-triple PD3 exhibits a monopole-like defect-mode shape at 59.94 kHz similar to that shown in Fig. 3b-i. Conversely, L-triple PD1 and L-triple PD2 exhibit in- and out-of-phase monopole-like defect-mode shapes at 59.92 and 59.96 kHz, similar to that shown in Fig. 3b-ii, while L-triple PD3 does not exhibit significant energy localization features. The corresponding defect-band frequencies increase by 70 Hz to 59.99 kHz, 60.01 kHz, and 60.03 kHz, when the electric circuit condition is switched to open-circuit.

### Piezoelectric energy harvesting performance

**Fig. 4:**
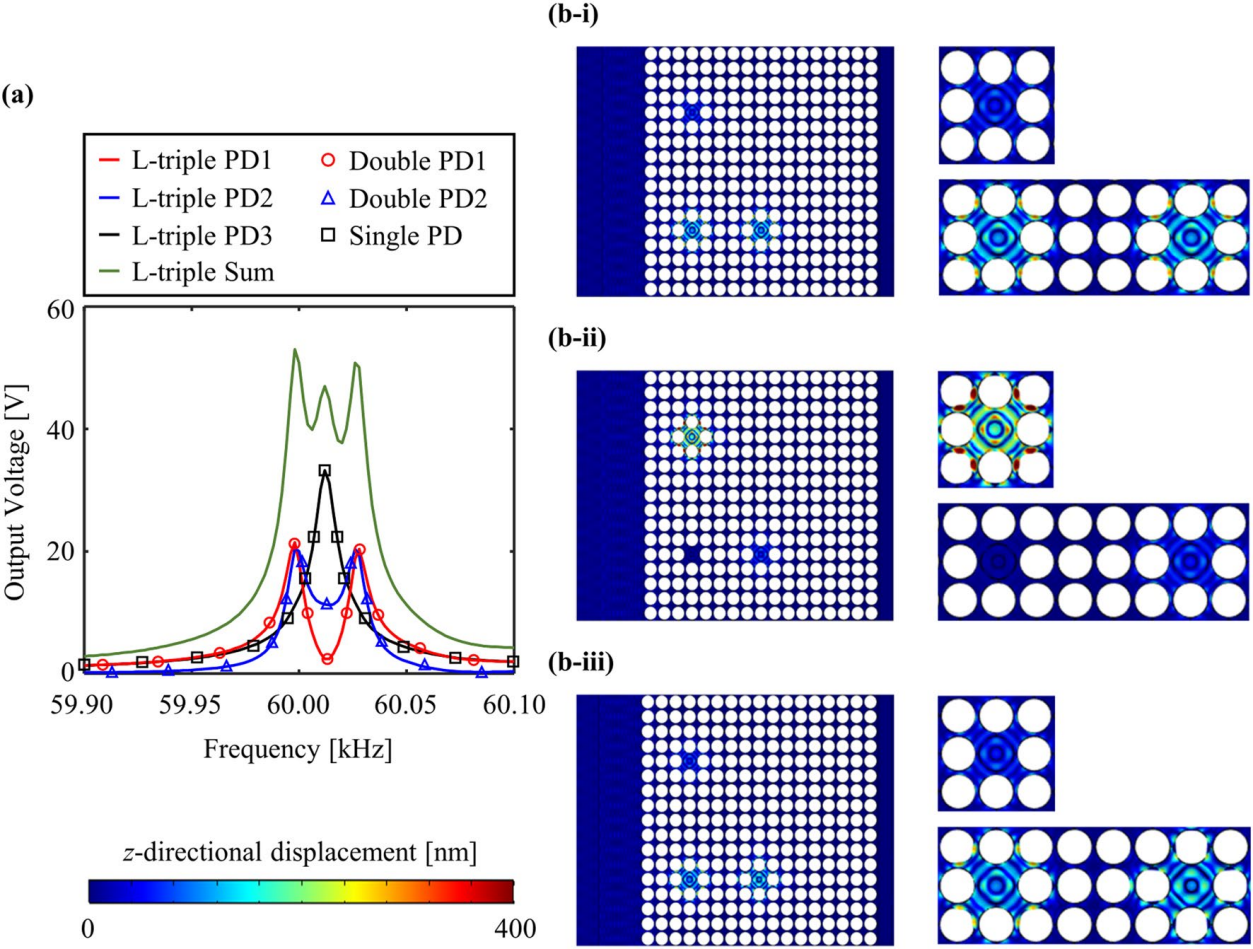
PEH performance results under open-circuit in the time-harmonic analysis: **a** output voltage FRFs, *z*-directional displacement fields of the proposed PnC design at **b**-**i** 59.99, **b**-**ii** 60.01, and **b**-**iii** 60.03 kHz

Energy localization and harvesting performance is evaluated in time-harmonic analysis. Analogous to the previous study [40], it is assumed that A_0_ Lamb waves with the displacement amplitude of 20 nm enter the systems over the frequencies. Similarly, the loss factor of the structures is set as 0.0001. Figure 4a presents output voltage FRFs under open-circuit. The output performance of the PEH systems with single and double defects is investigated similar to that of the previous section. Solid lines with red, blue, and black indicate PEH performance obtained from L-triple PD1, L-triple PD2, and L-triple PD3. The black squares indicate the results of the single-defect-incorporated PnC (Single PD). The red circles and blue triangles indicate the results of the double-defect-incorporated PnC (Double PD1 and Double PD2). Figure 4b-i to b-iii visualize the top-viewed *z*-directional displacement fields at the peak frequencies of 59.99, 60.01, and 60.03 kHz, respectively, when incident A_0_ Lamb waves enter from the left end of the large plate. Note that the output voltage value without the PnC is 0.5 V.

Two observations can be obtained from Fig. 4. First, the output voltage FRF obtained from Single PD coincides with that obtained from L-triple PD3. Similarly, the output voltage FRFs obtained from Double PD1 and Double PD2 match with those obtained from L-triple PD1 and L-triple PD2. Although triple defects are introduced, three peaks are not observed in each FRF. In contrast, only one peak frequency of 60.01 kHz is observed in the FRF of L-triple PD3, and two peak frequencies of 59.99 and 60.03 kHz are observed in the FRFs of L-triple PD1 and L-triple PD2. The output voltage values of the proposed PnC at each peak frequency are summarized in Table 2. Second, only L-triple PD3 exhibits significant amplification of the displacement fields at 60.01 kHz (Fig. 4b-ii), which vibrates in the monopole-like defect-mode shape. Conversely, at 59.99 kHz (Fig. 4b-i) and 60.03 kHz (Fig. 4b-iii), only L-triple PD1 and L-triple PD2 exhibit displacement amplification of the synchronously vibrating in- and out-of-phase monopole-like mode shapes. Therefore, it can be concluded that L-triple PD3 is effectively decoupled from the group of L-triple PD1 and L-triple PD2 during the time-harmonic analysis. The comparison between Figs. 3 and 4b-i to b-iii depicts that energy-localized behaviors vaguely appear in other piezoelectric defects at each peak frequency. The differences between Figs. 3 and 4 are similar to that of the differences observed in the vibrations between the normal mode shape during the modal analysis and operating deflection shape during the time-harmonic analysis.

**Table 2:** Output voltage values at peak frequencies and their sums under open-circuit

Defect	Value		
	59.99 kHz	60.01 kHz	60.03 kHz
L-triple PD1	20.44 V	11.22 V	20.39 V
L-triple PD2	21.46 V	2.40 V	20.36 V
L-triple PD3	11.13 V	33.24 V	10.36 V
Sum	53.03 V	46.8 V	51.11 V

**Fig. 5:**
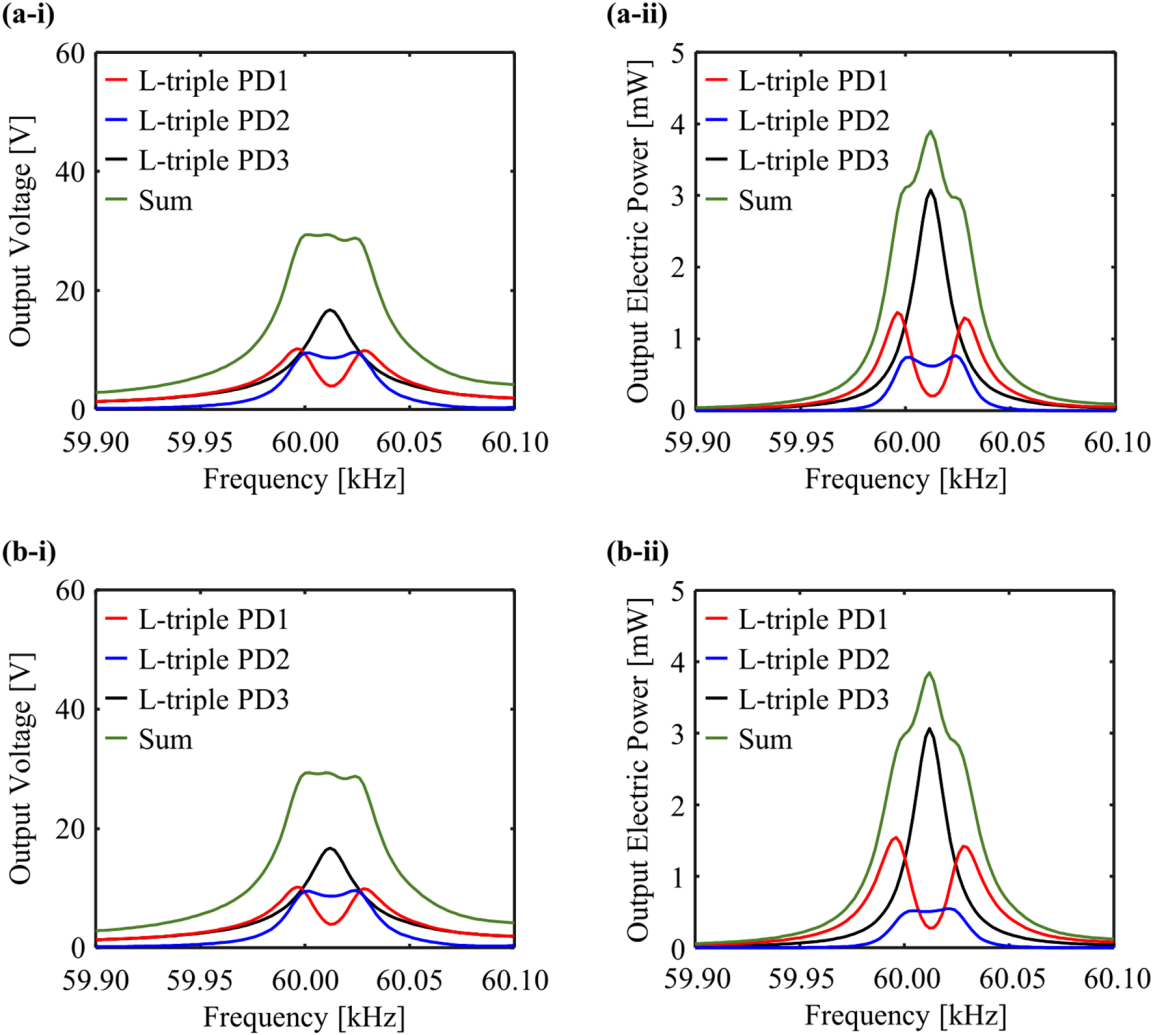
PEH performance results across the power-optimal electrical resistances in time-harmonic analysis: **a**-**i** output voltage FRFs and **a**-**ii** output electric power FRFs in Case I, **b**-**i** output voltage FRFs and **b**-**ii** output electric power FRFs in Case II

The power-optimal electrical resistances of L-triple PD3, and the group of L-triple PD1 and L-triple PD2 are independently calculated using the superposition property. The power-optimal electrical resistance for the maximum power generation can be numerically obtained at the corresponding peak frequency of each defect under open-circuit by sweeping the electrical resistance in the range of 0–100 MΩ [54, 55]. The power-optimal electrical resistance is calculated as 91.2 kΩ at the single peak frequency of 60.01 kHz for the isolated L-triple PD3. Subsequently, the power-optimal electrical resistance set of L-triple PD1 and L-triple PD2 at each peak frequency of 59.99 and 60.03 kHz is denoted as Cases I and II, respectively. Their power-optimal electrical resistances are numerically calculated as: Case I—(75.9 kΩ, 120.2 kΩ) at 59.99 kHz and Case II—(45.7 kΩ, 125.9 kΩ) at 60.03 kHz. Figure 5 depicts the two FRFs calculated for output voltage and electric power in each case. Table 3 summarizes the values of output electric power and voltage at each peak frequency.

**Table 3:** Output voltage and electric power values at peak frequencies and their sums across the power-optimal electrical resistances

Power-optimal electrical resistance	Defect	Quantity	Value		
			59.99 kHz	60.01 kHz	60.03 kHz
Case I	L-triple PD1	Voltage	10.2 V	3.93 V	9.91 V
		Electric power	1.37 mW	0.20 mW	1.22 mW
	L-triple PD2	Voltage	9.44 V	8.66 V	9.61 V
		Electric power	0.74 mW	0.62 mW	0.76 mW
	L-triple PD3	Voltage	9.58 V	16.8 V	8.73 V
		Electric power	1.00 mW	3.09 mW	0.84 mW
	Sum	Voltage	29.2 V	29.4 V	28.0 V
		Electric power	3.12 mW	3.92 mW	2.82 mW
Case I	L-triple PD1	Voltage	8.40 V	3.53 V	8.02 V
		Electric power	1.54 mW	0.27 mW	1.51 mW
	L-triple PD2	Voltage	6.83 V	8.00 V	6.42 V
		Electric power	0.37 mW	0.51 mW	0.53 mW
	L-triple PD3	Voltage	9.58 V	16.8 V	8.73 V
		Electric power	1.00 mW	3.09 mW	0.84 mW
	Sum	Voltage	24.8 V	28.3 V	23.2 V
		Electric power	2.92 mW	3.88 mW	2.87 mW

The output sinusoidal electric signals are transformed into closely constant electric signals using the AC-DC converter. Hence, the PEH performance generated from the three piezoelectric discs is summed. The green solid lines in Figs. 4 and 5 indicate the summed PEH performance. It can be observed that the proposed design concept can effectively widen the bandwidth for PEH and enhance the power generation. The principle of these results can be observed in the complementary features of PEH performance of the single (L-triple PD3) and double (L-triple PD1 and L-triple PD2) defects. A single-defect-incorporated PEH system enables higher output electric power at a single defect-band frequency, but with a narrow bandwidth. Conversely, a double-defect-incorporated PEH system can achieve wider (almost doubled) bandwidth owing to the defect-band splitting phenomenon. However, analogous to anti-resonance in a dynamic vibration absorber system, the PEH performance rapidly decreases between the two peak frequencies. The advantages and limitations of the single-defect-incorporated PEH system are opposite to those of the double-defect-incorporated PEH system. The output performance of the single defect can compensate for the output performance of the double defects in the proposed PnC design, which can significantly decrease between two peak frequencies. Conversely, the defect-band splitting of the double defects can compensate for the narrow bandwidth of the single defects. Hence, the proposed design broadens the bandwidth for elastic wave localization and ensures energy harvesting by minimizing the limitations of single and double defects.

## Conclusions

This study proposed a phononic crystal (PnC) design with L-shape triple defects to broaden the bandwidth for piezoelectric energy harvesting (PEH) under A_0_ Lamb waves. The unit cell used a square lattice of aluminum, wherein a circular hole was drilled to the center. The triple defects were introduced at (4, 5), (9, 5), and (4, 11) while arranging 17 × 17 unit cells such that holes were not perforated within the three unit cells. The defects at (4, 5) and (9, 5) were positioned at the bottom vertices of the letter ‘L’ while the remaining defect at (4, 11) was positioned at the top vertex in the top view. A piezoelectric disc, which was connected to an alternating-current-to-direct-current converter and the electrical resistance for power generation, adhered to the center of the top surface of each defect.

The basic principle of broadband PEH was the superimposition of two different PnC-inspired PEH systems into one system. The preliminary studies implied that five unit cells placed between the double defects were sufficient to represent the complete decoupling phenomenon. Hence, the well-isolated piezoelectric defect at (4, 11) behaved as a single-defect system. Conversely, the coupled piezoelectric defects at (4, 5) and (9, 5) behaved as a double-defect system. The piezoelectric defect at (4, 11) presented only one peak frequency of 60.01 kHz, while the remaining two piezoelectric defects presented two peak frequencies of 59.99 and 60.03 kHz. It was confirmed that the single defect compensated for the limitations of the double-defect-incorporated PEH system, wherein the performance significantly decreased between the two peaks, and the double defects compensated for the limitation of the narrow bandwidth of the single-defect-incorporated system. Therefore, the PnC with L-shape triple defects was proven valid for broadband energy localization and harvesting.

It should be noted that in the proposed design, although the L-shape arrangement of the triple defects is maintained, inter-distances among the defects depend upon the *Q*-factor (sharpness) near each peak frequency. If the *Q*-factor is large, the double defects should be relatively distant from each other to ensure that one peak frequency of the single defect and two peak frequencies of the double defects are adjacent. On the contrary, when the *Q*-factor decreases, the double defects should be closely located to ensure that the three peak frequencies are apart. Therefore, it is desirable to check the *Q*-factor at the material or structure levels in advance and perform parametric studies to specify the defect locations.

Lowering the target defect-band frequency is also an essential issue in this research area. As the lattice constant of the unit cell increases, the defect-band frequency, as well as the band gap, can decrease [56]. Alternatively, the stub-type unit cell enables to lower the target frequency range [57]. In addition, unlike in this study, the local resonance-type unit cell has abilities to drop the band gap [58]. In the future, the research on further lower-frequency regions can be explored.

## Data Availability

The datasets used and/or analyzed during the current study are available from the corresponding author on reasonable request.
